# SPECIES IDENTIFICATION OF *CANDIDA* ISOLATES OBTAINED FROM ORAL LESIONS OF HIV INFECTED PATIENTS

**DOI:** 10.4103/0019-5154.57622

**Published:** 2009

**Authors:** V P Baradkar, S Kumar

**Affiliations:** *From the Department of Microbiology, Lokmanya Tilak Municipal Medical College & General Hospital, Sion, Mumbai - 400 002, India.*

**Keywords:** *Oral candidiasis*, *HIV*, *Candida dubliniensis*, *Candida species*

## Abstract

A total of 60 patients suspected to have AIDS with oral lesions suggestive of oral candidiasis were studied. *Candida species* were isolated from 50 patients. *Candida albicans* was the commonest isolate (70 %) followed *Candida parapsilosis*(15%), *Candida glabrata* (7.5%) and *Candida tropicalis* (5%) respectively. *Candida dubliniensis* was isolated from a single case only. Though the reports from developed countries show more prevalence of the novel species *Candida dubliniensis*, in our study it was isolated in a single case. All the patients were treated successfully with oral fluconazole for 7 days except for the patients from which *Candida glabrata* was isolated, who were treated with Amphotericin B.

## Introduction

Oral candidiasis is the most common opportunistic infection in patients with HIV infection in India.[1] Various studies have shown increasing incidence of candidiasis in HIV infected persons.[2–5] In the recent past, there have been increasing reports implicating the non-albicans species of *Candida* in oropharyngeal candidiasis.[2–5] We studied the prevalence and clinical and microbiological variations of oral candidiasis in HIV infected persons.

**Figure 1:**
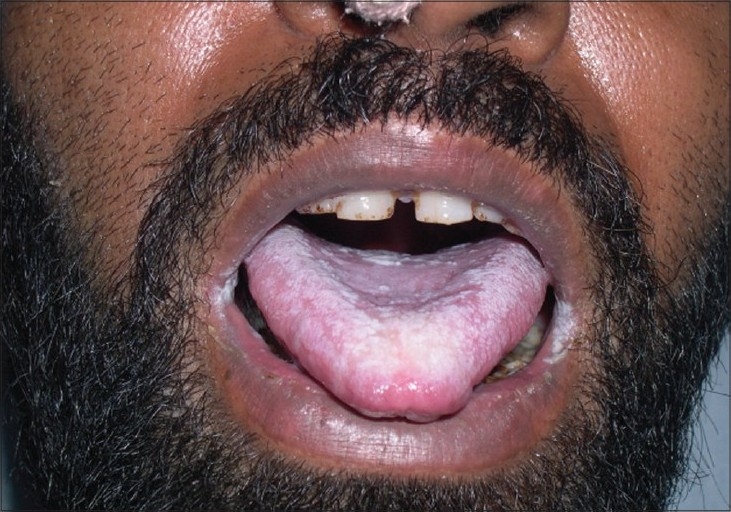
Clinical photograph showing typical presentation of oral thrush (white curdy patches in dorsum of tongue)

**Figure 2:**
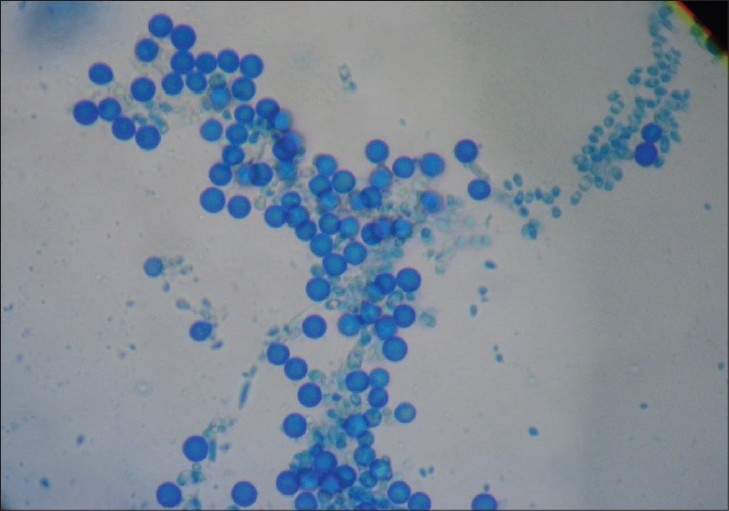
LPCB mount of colonies on tobacco agar showing clustering of chlamydospores at the tip of short pseudohyphae suggestive of *Candida dubliniensis*

The study was conducted in the Department of Microbiology, Lokmanya Tilak Municipal Medical College and General Hospital, Mumbai, from July 2005 to June 2008. A total number of 61 HIV seropositive patients with oral lesion/lesions suggestive of oral candidiasis, which were referred from the Department of Medicine and Dermatology, were included in the study of which 46 were males and 15 were females (male: female ratio of 3:1). The age group ranged between 20 and 60 years (mean age 40 years). Most common clinical presentation was pseudomembranous lesions [Figure 1] in 50 cases, followed by erythematous lesions in 10 cases. *Candida* species were isolated from 40 patients, demonstrating a prevalence of 66.57%. All the patients from whom *Candida* species were isolated presented with a burning sensation, dysphagia, and odynophagia suggestive of esophageal involvement. *Candida albicans* was the most common isolate 70% (28/40), followed by *C. parapsilosis* 15% (6/40), *Candida tropicalis* 5% (2/40), and *C. glabrata* 7.5% (3/40), while *C. dubliniensis* [Figure 2] was isolated in a single case (2.5%). Oropharyngeal candidiasis, the most common opportunistic infection in patients with HIV, occurs in as many as 90% of HIV infected persons at some point during the course of disease.[6] The prevalence of oral candidiasis in HIV infected patients in India varies between 41%[4] and 85%.[1] In India, it is the most common manifestation in HIV infected persons.[1–5] Though *C. albicans* is the most common cause of oral candidiasis, certain non-albicans species such as *C. parapsilosis, C. glabrata, C. tropicalis, C. kefyr* and new species *C. dubliniensis*[7] are now encountered. This study shows substantial increase in isolation of non-*Candida albicans* species (30%) as reported earlier by Kaviarasan *et al*.[1] (20.2%), and the new species i.e. *C. dubliniensis* is also isolated from HIV seropositive cases in this study.
